# Exploration of related factors of suicide ideation in hospitalized older adults

**DOI:** 10.1186/s12877-023-04478-w

**Published:** 2023-11-16

**Authors:** Su-Jung Liao, Yu-Wen Fang, Tse-Tsung Liu

**Affiliations:** 1 Department of Nursing, College of Nursing, Tzu Chi University of Science and Technology, Hualien, Taiwan; 2 Department of Family Physician and Geriatrician, Mennonite Christian Hospital, Hualien, Taiwan

**Keywords:** Suicidal ideation (SI), Older adults hospitalized patients, Mental health status, Quality of life, Depressive mood

## Abstract

**Background:**

With the rapid aging of the population structure, and the suicide ideation rate also increasing year by year, the ratio of people over 65 to the total number of deaths is increasing yearly. The study provides a reference for researchers interested in older adults’ care to explore SI further affecting older adults in the future and provide a reference for qualitative research methods or interventional measures.

**Objective:**

The objective of this study is to explore the influence of mental health status, life satisfaction, and depression status on suicidal ideation (SI) among hospitalized older adults.

**Methods:**

In a cross-sectional correlation study, taking inpatients over 65 years old in a regional teaching hospital in eastern Taiwan, and the BSRS-5 ≧ 5 points of the screening cases, a total of 228 older adults agree to conduct data analysis in this study. Mainly explore the influence of personal characteristics, mental health status, life satisfaction, and depressed mood on SI among the hospitalized older adults. The basic attributes of the cases used in the data, mental health status, cognitive function, quality of life, depression, and suicide ideation, the data obtained were statistically analyzed with SPSS 20/Windows, and the descriptive statistics were average, standard deviation, percentage, median, etc. In the part of inference statistics, independent sample t-test, single-factor analysis of variance, Pearson performance difference correlation, and logistic regression analysis were used to detect important predictors of SI.

**Results:**

Research results in (1) 89.5% of hospitalized older adults have a tendency to depression. 2.26.3% of the older adults had SI. (2) Here are significant differences in the scores of SI among hospitalized older adults in different economic status groups and marital status groups. (3) The age, marital status, and quality of life of the hospitalized older adults were negatively correlated with SI; economic status, self-conscious health, mental health, and depression were positively correlated with SI. (4) The results of the mental health status and SI is (r = .345, *p* < .001), higher the score on the BSRS-5 scale, the higher the SI. The correlation between the depression scale score (SDS-SF) and SI was (r = .150, *p* < .05), the higher the depression scale score, the higher the SI.

**Conclusion:**

The results of the study found that there was a statistically significant correlation between SI in older adults and age, marital status, economic status, mental health, quality of life, and depression, and also showed that they might interact with each other; the older adults in BSRS-5, GDS-SF, quality of life scale scores have statistically significant differences as essential predictors of SI. The results of this study suggest that medical staff can use the BSRS-5 scale to quickly screen and evaluate the mental health status of older adults, hoping to detect early and provide preventive measures, thereby improving the quality of life of older adults.

**Supplementary Information:**

The online version contains supplementary material available at 10.1186/s12877-023-04478-w.

## Introduction

In Taiwan, affected by the rapid aging of the population structure, the ratio of people over 65 to the total number of deaths is increasing yearly [1–3]. In 2021, the number of suicide deaths among the older adults over 65 will be 27.6 per 100,000 population, accounting for 29.7% of the total death rate [4]. The associated risk factors and prevalence of suicidal behaviors are diverse and are closely related to settings, measures, age groups, and different populations [5, 6]. Compared with younger people, older adults are at a higher risk of suicide in most countries [7–9]. The prevalence of SI varies among the older adults, ranging from 0.7% in older adults’ primary care patients to 26% in acute medically ill older adults’ inpatients [3, 10]. Furthermore, there are other risk factors related to SI and suicidal behaviors, which include living alone [11, 12], financial problems [3, 12], marital status [13, 14], poor perception of health [5, 12], poor social support [6, 12, 15, 16], and chronic physical illness [6, 15, 17, 18], impaired cognition [6, 19–21], clinical depression [12, 14, 16, 22–24]. Several studies have revealed multi-domain factors related to SI or suicidal behaviors. Quality of life (QOL) has been found to be associated with the risk of SI or suicidal behaviors in the older adults [25–27].

More attention should be paid to older adult’s inpatients with physical illness because they are more likely to have a suicide attempt and suicide death because of old age, the burden of physical diseases, and an increased concurrence of depression [22, 23, 28, 29]. With a growing older adults’ population in many countries, late-life depression is an increasing challenge [18, 30–33]. Thus, singling out older adult’s inpatients with SI at an early stage and providing timely adequate treatment in a general hospital, may decrease rates of suicidal behaviors and related mortality [10, 34–36].

Although this issue is essential, only a few studies have explored the prevalence and relevant factors of Suicide Ideation in older adult’s inpatients with hospitalized older adult’s patients [3, 23]. Thus, the purpose of the study is to explore the correlation between personal characteristics, mental health status, quality of life, depressed mood, and SI in hospitalized older adult’s patients.

## Methods

### Procedure

**Fig. 1:**
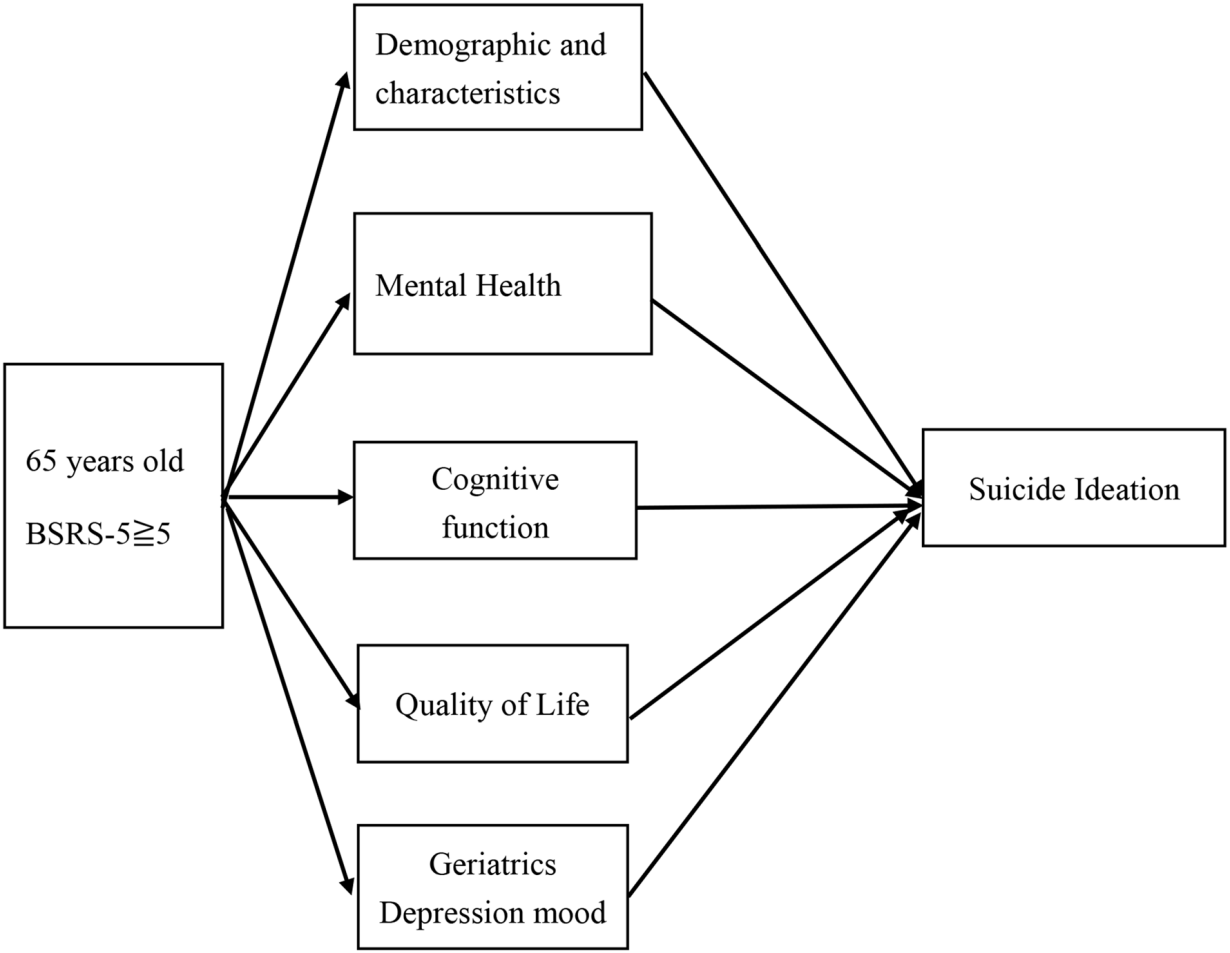
Research Framework

A cross-sectional design was used to describe mental health, cognitive function, quality of life, depression, and SI (SI), among hospitalized older adults in eastern Taiwan. Data collection started in January 2018 and ended in December 2018. The researchers conducted one-on-one explanations and questionnaires to the participants. This study explores the influence and predictive power of older adult’s demographic characteristics, mental health, cognitive function, quality of life, and depressed mood on suicidal ideation (Fig. 1).

### Participants

The participants were recruited inpatients over 65 years old in a regional teaching hospital in eastern Taiwan as the research object, and data collection procedures were conducted through inpatient centers to assess BSRS-5 ≧ 5, a total of 228 older adults agreed to conduct data analysis in this study, inclusion criteria: (1) above 65 years of age; (2) Brief Symptom Rating Scale (BSRS-5) > 5; (3) SI of Brief Symptom Rating Scale (BSRS-5)>1; (4) Mini-Mental State Examination (MMSE) > 20; (5) clear consciousness and attention sustainability of, at least, 20 min; (6) depressed mood or symptoms do not interfere with interviews and data collection; (7) volunteering to participate in this study. Sample exclusion criteria were: (1) visual, hearing, and communication impairments; (2) alcohol or drug dependence. Data collection procedures are conducted through inpatient centers to assess BSRS-5 ≧ 5, visiting subjects in hospital wards, explain the purpose of the research, and obtain consent from the subjects before collecting data. The sample estimate is based on three times the number of questions in the questionnaire. There are 68 questions in total, and 204 cases need to be collected. Adding a 15% attrition rate, it is estimated that 234 cases will be collected, and the final number of cases will be 228 [36].

### Measures

#### Demographic and medical characteristics

Older adult’s characteristics include gender, age, education level, living status, economic status, marital status, and perceived health, by a research assistant through face-to-face interviews using a questionnaire developed by the researchers.

#### BSRS-5 (Brief symptom rating scale)

This scale was first introduced by Lung [37], and developed by Professor Mingbin Li from the Department of Psychiatry, College of Medicine, National Taiwan University School (2003) [38]. Its reliability and validity showed good internal consistency through analysis of different ethnic groups (Cronbach’s α = 0.77–0.90), the test-retest reliability is 0.82 [38]. The BSRS-5 measures the five symptom items of anxiety, depression, hostility, interpersonal sensitivity/inferiority, and insomnia and contains five items selected from the BSRS-50 that were highly correlated with each other, and included an additional question about suicide ideation (SI). Specifically, the BSRS-5 measured the following symptoms: difficultly in falling asleep, experiencing annoyance or anger, feeling down or depressed, a sense of inferiority to others, and having suicidal thoughts. The total score of the BSRS-5 is 24 points, with 0–4 points for each item (0: Not present, 1: Slight, 2: Moderate, 3: Severe, 4: Extremely severe). A variable of a total score of BSRS-5 greater than 5 was formed because it represented significant psychiatric morbidity for an individual, and it was highly associated with suicidal behavior in older adults [22, 23, 39]. The internal consistency of Cronbach’s α was 0.83 in this study.

#### Mini-mental status examination (MMSE)

Cognitive function was assessed based on a Chinese version [40] of the Mini-Mental State Examination (MMSE) [41], in which the maximum score is 30, and a higher score indicates better cognition. It is divided into 11 items, including: time recognition, place recognition, short-term memory, attention and calculation, memory check of recent things, object names, repeating what others say, understanding what others say, and understanding the meaning of words or pictures, write sentences and draw graphics. The test-retest reliability was 0.89 and the interrater agreement was 0.83 [40]. Impaired cognition was defined as those who were uneducated and had MMSE scores less than 14, and the educated with scores less than 24 [41]. The internal consistency Cronbach’s α was 0.80 in this study.

#### World health organization quality of life-BREF (WHOQOL-BREF TW)

To measure the QOL, the Taiwanese version of the World Health Organization Quality of Life-BREF (WHOQOL-BREF TW) [42] was used to assess the global QOL of patients in medical settings. The WHOQOL-BREF TW includes 26 items (24 items that represent each of the 24 specific facets of the WHOQOL-100 and 2 global/general items). The internal consistency (Cronbach’s α) were 0.70 to 0.77 [42]. In addition, in the WHOQOL-BREF TW, two additional national items were generated and validated from the Taiwan version of the WHOQOL-100 [43]. The factor structure of the WHOQOL-BREF TW includes 4 domains, i.e., physical (QOL-PHY), psychological (QOL-PSY), social relationships (QOL-SR), and environmental (QOL-ENV). For a given item or domain of the WHOQOL-BREF, a higher score indicates a greater level of quality of life. The internal consistency Cronbach’s α was 0.86 in this study.

#### Geriatric depression scale short form, (GDS-SF)

The shortened form (GDS-S) is comprised of 15 items chosen from the Geriatric Depression Scale-Long Form (GDS-L). Its reliability and validity showed good internal consistency (Cronbach’s α = 0.43–0.89), the test-retest reliability is 0.84 [44]. These 15 items were chosen because of their high correlation with depressive symptoms in previous validation studies [44]. Of the 15 items, 10 indicate the presence of depression when answered positively while the other 5 are indicative of depression when answered negatively. This form can be completed in approximately 5 to 7 min, making it ideal for people who are easily fatigued or are limited in their ability to concentrate for longer periods of time [45, 46]. The internal consistency of Cronbach’s α was 0.76 in this study.

#### Beck scale for suicide ideation; BSS

Suicide ideation was measured using the Beck Scale for SI (BSS) [47, 48], the BSS contains 21 statement groups each assessing various aspects of suicidal ideation. The internal consistency (Cronbach’s α) was 0.89, the interrater agreement was 0.83 [47, 48]. Each statement group consists of three sentences that describe different intensities of suicidal ideation, representing a three-point scale (0 to 2) [49]. Item 1 of the BSS presents participants with three statements: (0) “I have no desire to kill myself”, (1) “I have a weak desire to kill myself” or (2) “I have a moderate to the strong desire to kill myself”. A total composite score is calculated by summing the numeric values of the endorsed statements. Total scores on the BSS range from 0 to 38, with greater scores indicating greater suicide ideation [49]. The internal consistency of Cronbach’s α was 0.84 in this study.

#### Data analyses

This study uses SPSS 20. Windows for statistical analysis of data, using methods such as mean, standard deviation, percentage, median, etc. in the part of descriptive statistics, and using t-test, Pearson poor performance correlation, and logistic regression analysis methods.

Category variables in the demographic variable data include gender, age, education level, living status, economic status, marital, perceived health status, etc., and are analyzed by “frequency distribution” and “percentage”. Continuous variables in the demographic variable data include age, which are analyzed by “mean” and “standard deviation” and classified, and analyzed by “frequency distribution” and “percentage”. BSRS-5, MMSE, WHOQOL-BREF, BSS, GDS-SF, the scoring situation is analyzed using “total score”, “mean”, and “standard deviation”. The categorical variables in the demographic variable data include gender, age, education level, living status, economic status, marital, perceived health status, etc., and the correlation with the BSS, using independent sample t-test and single factor variation analysis. Demographic variables, BSRS-5, MMSE, WHOQOL-BREF, BSS, and GDS-SF scores were analyzed using Pearson product-moment correlation coefficient and Spearman’s rank correlation. Prediction of suicidal ideation by demographic variables, BSRS-5, WHOQOL-BREF, BSS, GDS-SF, etc. using Logistic regression analysis.

#### Ethics approval

Before data collection, Institutional Review Board approval was obtained, by Mennonite Christian Hospital Ethics Committee (MCH-IRB-14-11-019). Further, informed consent was obtained from the participants after explaining the study purpose and procedures and the participants’ rights and after assuring the participants that all collected data would be kept private and confidential. Data was kept confidential and coded on a password-protected computer in the principal investigator’s office.

## Results

### Participants

The average age for all participants (n = 228) was 75.2 (shown in Table 1). Male patients accounted for 50.4% (n = 115); education level is mostly literate:101 (44.3%); 114 people with a spouse (50%); live with their spouse and children: 117 people (51.3%); there are 152 people (66.7%) who feel that their economic situation is “fair”, and 60 people (26.3%) think it is “not enough”; felt that their health status was “bad”: 171 (75%).

**Table 1:** Describing the personal characteristics of the hospitalized older adults. (*N* = 228)

Variable	Characteristics	n	%
Gender	Male	115	50.44
	Female	113	49.56
Age	65 ~ 74	116	50.88
	75 ~ 84	81	35.52
	85 year old above	31	13.60
Education level	Recognize words	101	44.30
	Elementary school	50	21.93
	Junior high school	38	16.67
	Senior high school above	39	17.10
Living Status	Alone	54	23.68
	With spouse	57	25.00
	With spouse and children	117	51.32
Economic Status	Ample	16	7.02
	Passable	152	66.66
	Insufficient	60	26.32
Marital	No spouse	114	50.00
	Married	114	50.00
Perceived health	Very bad	12	5.26
	Not good	171	75.00
	Good	45	19.73

### BSRS-5 (Brief symptom rating scale)

The average score of BSRS-5 was 7.84 (SD = 2.30) (shown in Table 2), 69.7% of the cases were “trouble falling asleep” (n = 159); 54.3% were “feeling tense or keyed up” (n = 124); 60.2% (n = 164); “feeling blue” accounted for 79.4% (n = 181). According to the severity of the health problems, “feeling blue” (79.4%), " trouble falling asleep” (69.7%), “feeling easily annoyed or irritated” (60.2%), “feeling tense or keyed up” (54.3%), “feeling inferior to others” (22.8%). 60 people had suicidal thoughts, accounting for 26.3%.

**Table 2:** The distribution of mental health status (BSRS-5) scale. (N = 228)

Variable	n	%
Normal (< 6 points)	11	4.8
Mild emotional distress (6–9 points)	175	76.8
Moderate emotional distress (10–14 points)	38	16.7
Severe emotional distress (more than 15 points)	4	1.8
Having suicidal thoughts	60	26.3

### Mini-mental status examination (MMSE)

The average cognitive function score of the hospitalized older adults was 22.73 points (SD = 4.30), of which 206 people had normal cognitive function, accounting for 90.4%, followed by mild cognitive impairment, 22 people accounted for 9.6%. Satisfaction with the quality of life of older adults, the average score of the overall quality of life is 48.56 (SD = 5.91); the overall quality of life is mostly moderately satisfied, accounting for 63.2% (n = 144), and 70.6% (n = 161) are dissatisfied in terms of health satisfaction. Comparing the weighted scores of the four quality of life categories, the lowest is the average value of the physiological category of 11.22 points (SD = 2.08); the second is the average value of the psychological category of 11.55 points (SD = 1.91); the average value of the social relationship category is 12.75 (SD = 2.00); The highest score is the average of 13.04 points (SD = 2.02) in the environmental category.

### Geriatric depression scale short form, (GDS-SF)

The GDS-SF was used to detect the depression status of the hospitalized older adults, and the score ranged from 2 to 14 points (the score range was 0 to 15 points), with an average score of 8.21 points (SD = 2.86); among them, mild depression was mostly from 5 to 9 points There were 114 people (accounting for 50%); followed by 90 people (accounting for 39.5%) with moderate to severe depression ranging from 10 to 15 points. In this study, 89.5% of hospitalized older adults were prone to depression.

### Beck scale for suicide ideation; BSS

The average total score of BSS research results for questions 1 to 19 is 2.29 (SD = 4.62) (shown in Table 3). For the critical value of SI, as long as there is a response to the fourth and fifth questions of the scale, it is considered to have SI. A total of 60 people have SI and then answer questions 6–19. In the first question “Have the will to live” there are 22 people (9.6%) who have a weak will to live, and there are 5 (2.2%) older adults who do not have a weak will to live; in the second question “have the will to die” 47 (20.6%) had a weak desire to die, and 13 (5.7%) had a strong desire to die; 15 people (6.6%) had roughly the same reasons, and 6 (2.6%) of the older adults had the reason to die better than to survive; 51 people (22.4%) wanted to commit suicide in question 4 “Active suicide attempt”, and there are 9 (3.9%) older adults who have a strong desire to commit suicide; in question 5 “Passive suicide attempts”, if they find their lives are threatened, 16 people (7.0%) will let life and death be resigned to fate, and Four (1.8%) older adults would not take the necessary steps to avoid death if they found their life threatening.

**Table 3:** Suicide ideation scale in hospitalized older adults. (N = 228)

Scale Variable	n	*M (SD)*	0分	1	2
			*n* (%)	*n* (%)	*n* (%)
1.Wish to live *	228	0.14(0.40)	201(88.2)	22(9.6)	5(2.2)
2.Wish to die*	228	0.32(0.57)	168(73.7)	47(20.6)	13(5.7)
3.Reasons for living or dying	228	0.12(0.39)	207(90.8)	15(6.6)	6(2.6)
4.Active suicide attempt	228	0.30(0.54)	168(73.7)	51(22.4)	9(3.9)
5.Passive suicide attempt	228	0.11(0.36)	208(91.2)	16(7.0)	4(1.8)
6.Duration of suicidal thoughts	60	0.18(0.43)	50(83.3)	9(15.0)	1(1.7)
7.Frquency of ideation	60	0.10(0.35)	55(91.7)	4(6.6)	1(1.7)
8.Attitude toward ideation	60	0.62(0.61)	27(45.0)	29(48.3)	4(6.7)
9.Control over suicidal action	60	0.82(0.50)	14(23.3)	43(71.7)	3(5.0)
10.Deterrents to attempt	60	0.77(0.59)	19(31.7)	36(60.0)	5(8.3)
11.Reasons for attempt	60	0.75(0.70)	24(40.0)	27(45.0)	9(15.0)
12.Specificity of planning	60	0.13(0.34)	52(86.7)	8(13.3)	0(0)
13.Availability or opportunity	60	0.18(0.53)	53(88.3)	3(5.0)	4(6.7) )
14.Capability to carry out attempt	60	0.37(0.61)	42(70.0)	14(23.3)	4(6.7)
15.Expectancy of actual attempt	60	0.67(0.54)	22(36.7)	36(60.0)	2(3.3)
16.Extent of actual preparation	60	0.08(0.27)	55(91.7)	5(8.3)	0(0)
17.Suicide note	60	0.02(0.12)	59(25.9)	1(0.4)	0(0)
18.Final acts	60	0.08 (0.27)	55(91.7)	5(8.3)	0(0)
19.Deception and concealment	60	0.18(0.43)	50(83.3)	9(15.0	1(1.7)
**Total Score**	**228**	**2.29(4.62)**			
20.Frquency of suicide attempt	228	0.20(0.46)	189(82.9)	33(14.5)	6(2.6)
21.Suicide attempt and wish to die	228	0.64(0.77)	21(9.2)	11(4.8)	7(3.16)

Questions 20 and 21 are to find out whether the older adult subject has attempted suicide, and the severity of the attempted suicide at that time, to help medical staff understand the background characteristics of the subject, which are not included in the total score of the BSS. In terms of answering 20 questions, 33 (14.5%) of the older adults had attempted suicide once, and 6 (2.6%) had attempted suicide twice or more. In the 21st question, 11 people (4.8%) had a moderate desire to die in their last suicide attempt; 7 people (3.1%) had a high desire to die in their last suicide attempt.

### Related factors of SI

The results of the study found that the depression status was significantly correlated with educational level (F(3, 224) = 3.19, *p* = .025), perceived health status (F(2, 225) = 5.34, *p* = .005), and other variables among different groups (shown in Table 4). There is a significant difference, and after Scheffe’s Post-Hoc comparison, the depressive state score of the literate people is higher than that of the middle school older adults; the depression state score of those who feel that their health is very bad is higher than that of the older adults who feel that their health is good.

**Table 4:** Correlation between characteristics variables and depressive status in hospitalized older adults. (*N* = 228)

Variable	Characteristics	*n*	*M (SD*)	*t / F*	*P*	*Scheffe*
Gender	Male	115	8.32(2.93)	0.41	0.55	
	Female	113	8.10(2.79)			
Age	65 ~ 74	116	8.04(2.94)	0.78	0.45	
	75 ~ 84	81	8.53(2.74)			
	85 year old above	31	8.00(2.91)			
Education	1. Recognize words >	101	8.84(2.88)	3.19	0.025*	1>3
	2. Elementary school	50	7.76(2.89)			
	3. Junior high school	38	7.45(2.59)			
	4. Senior high school above	39	7.90(2.80)			
Living Status	Alone	54	8.35(2.78)	0.62	0.535	
	With spouse	57	7.84(2.78)			
	With spouse and children	117	8.32(2.95)			
Economic Status	Ample	16	7.00(2.89)	1.60	0.202	
	Passable	152	8.26(2.83)			
	Insufficient	60	8.42(2.92)			
Marital Status	No spouse	114	8.262.82)	0.27	0.78	
	Married	114	8.16(2.92)			
Perceived health	1. Very bad	12	10.08(2.57)	5.34	0.005**	1>3
	2. Not good	171	8.33(2.79)			
	3. Good	45	7.27(2.94)			

*p < .05, ** *p* < .01, *** *p* < .001

The study found that age, marital status, economic status, mental health status, depression status, physical health, psychological health, environmental health, and overall quality of life among the personal characteristics of the hospitalized older adults were significantly related to their SI; Personal characteristics age, marital status, physical health, psychological health, environmental health, overall quality of life score were negatively correlated with SI. Economic status, perceived health, mental health status, and depression status were positively correlated with SI (shown in Table 5). The older adults without a spouse have a higher correlation with SI than those with a spouse (r = − .159, *p* < .01); The lower the score in the physical health domain, the higher the SI (r = − .249, *p* < .001); the lower the psychological health domain score, the higher the SI (r = − .216, *p* < .001); the lower the environmental health domain score, the higher the SI (r = − .166, *p* < .01), the correlation coefficient between overall quality of life score and SI (r = − .217, *p* < .001), the lower the overall quality of life total score, the higher the SI. The correlation coefficient between economic status and SI is (r = .242, *p* < .001), the poorer the economic situation, the higher the correlation with SI, perceived health and SI is (r = .131, *p* < .05), the worse the perceived health, the higher the correlation with SI. The mental health status and SI is (r = .345, *p* < .001), the higher the score on the BSRS-5 scale, the higher the SI. The correlation between the depression scale score (SDS-SF) and SI was (r = .150, *p* < .05), the higher the depression scale score, the higher the SI.

**Table 5:** Correlation between demographic characteristics, mental health status, quality of life status, depression status, and suicidal ideation status of hospitalized elderly adults. (*N* = 228)

	1	2	2	4	5	6	7	8	9	10	11	12	13	14	15	16
1. Suicidal ideation	1	− 0.109*	0.050	− 0.019	− 0.159**	0.242***	− 0.045	0.133^*^	0.345***	0.042	0.150*	− 0.249***	− 0.216***	− 0.166**	− 0.008	− 0.217***
2. Age		1	0.050	− 0.163*	− 0.181**	0.007	0.045	− 0.045	− 0.112*	− 0.328***	0.019	− 0.203***	− 0.006	0.058	0.008	− 0.051
3. Gender			1	− 0.347***	0.342***	0.031	0.188**	− 0.075	0.101	− 0.229***	− 0.039	0.031	0.009	− 0.016	0.183**	0.070
4. Education ^a^				1	0.167**	0.155**	− 0.104	0.071	− 0.044	0.433***	− 0.184**	0.119*	0.193**	0.193**	0.104	0.201**
5. Marital Status ^a^					1	− 0.167**	-158**	− 0.012	0.045	− 0.098	0.011	− 0.138*	− 0.049	− 0.083	− 0.060	− 0.122
6. Economic Status ^a^						1	− 0.049	0.169**	153*	− 0.050	0.084	− 0.201***	− 0.279***	− 0.412***	0.000	− 0.291***
7. Living Status ^a^							1	0.028	0.034	− 0.193**	0.024	− 0.017	0.003	− 0.045	0.229***	0.051
8. Perceived health ^a^								1	0.221***	− 0.044	0.186**	− 0.412***	− 0.280***	− 0.152*	− 0.015	− 0.291***
9. Mental health									1	− 0.041	0.183**	− 0.191**	− 0.189**	− 0.113*	0.005	− 0.166*
10. Cognitive function										1	− 0.282***	0.323**	0.335***	0.249***	0.150*	0.359***
11. Depressive mood											1	− 0.375***	− 0.637***	− 0.359***	− 0.407***	− 0.600***
12. Physical health												1	0.499***	0.432***	0.162**	0.717***
13. Mental Health													1	0.549***	0.447***	0.841***
14. Environmental Health														1	0.255***	0.760***
15. Social health															1	0.629***
16. 12-14Total Score																1

*p < .05, ** *p* < .01, *** *p* < .001, ^a^ : spearman rank correlation

## Discussion

The results of the study show that suicide ideation is related to age in hospitalized older adults, and the older the older adults, the higher the correlation with SI (r = − .109, *p*<0.05) [11, 12]. The hospitalized older adults found that those whose economic status was “insufficient” (M = 3.9, SD = 6.1) had higher SI scores than those whose economic status was “Passable” (M = 1.9, SD = 4.0) and “Ample” (M = 0.4, SD = 4.0), and there was a significant difference; shows that financial problems are a significant risk factor for SI in older adults (*p* = .003). There was a significant difference between the marital status and SI of the older adults, and the score of SI in the older adults without a spouse (M = 3.0, SD = 5.0) was higher than that of those with a spouse (M = 1.6, SD = 4.0) and the difference was significant (*p* = .017), Some studies have mentioned that widowed, single, and divorced older adults have a higher risk of suicide and suicide mortality, and divorce is an important factor, followed by widowhood [13, 14, 50]. There is a positive correlation coefficient between perceived health status and SI (r = .131, *p* < .05), the worse the perceived health is the higher the correlation with SI [6, 12].

BSRS-5 is a fairly effective detection tool for older adults suicide screening in Taiwan [12, 22, 23]. Mental health status is positively correlated with SI (r = .345, *p* < .001), the higher the mental health status score, the higher the SI, which is one of the predictors of SI in older adults, and an important factor for SI in the older adults [12, 14, 16, 22, 23]. The study used the GDS-SF to detect the depression status of the hospitalized older adults, the results of the study found that depression status was positively correlated with SI, and the correlation coefficient was (r = .150, *p* < .05). The higher the value, the higher the correlation with SI [12, 16, 22–24].

The SI was negatively correlated with quality of life physical health, psychological health, environmental health, and overall quality of life scores, and there was a significant difference. The correlation coefficient of physical health (r = − .249, *p* < .001); psychological health (r = − .216, *p* < .001); environmental health (r = − .166, *p* < .01); the total score of the overall quality of life (r = − .217, *p* < .001), the lower the quality of life score above, the higher the correlation with SI [6, 15, 17, 27], those who have SI have lower quality of life satisfaction [22, 23].

## Conclusion

The study found that 89.5% of hospitalized older adults have a tendency to depression. 26.3% of the older adults had SI. The score of SI among hospitalized older adults has obvious differences in personal traits, economic status, and marital status. Personal characteristic age, marital status, physical health category, mental health category, environmental health category, and overall quality of life total scores of the hospitalized older adults were negatively correlated with SI; economic status, perceived health, mental health status, depression status, and SI positively correlated. In the future, in addition to requiring a research intervention, study results will provide clinical and community mental health care professionals with a reference for care to improve the depressive symptoms of the older adults so that every older adult be able to live and enjoy a healthy life, both in body and mind.

The research sample was selected from old adults who were hospitalized due to illness. They may have physical pain and functional decline. During the interview, the impact on physical strength and concentration, the establishment of interpersonal relationships, trust in the external environment, and familiarity cannot reveal the true part of the heart. Therefore, the scores on each scale may be slightly underestimated.

### Relevance to clinical practice

The study found the seriousness of the mental health of the older adults. In addition to focusing on the physical needs of the older adults during the care process, medical staff also need to pay attention to the mental health of the older adults. The research results can provide a reference for researchers interested in older adults’ care to further explore SI affecting older adults in the future and provide a reference for qualitative research methods or interventional measures.

### Electronic supplementary material

Below is the link to the electronic supplementary material.

Supplementary Material 1

Supplementary Material 2

## Data Availability

The datasets generated and/or analyzed during the current study are not publicly available due to privacy / ethical requirements but are available from the corresponding author on reasonable request.
