# Correction: Exploration of related factors of suicide ideation in hospitalized older adults

**DOI:** 10.1186/s12877-024-04713-y

**Published:** 2024-03-06

**Authors:** Su-Jung Liao, Yu-Wen Fang, Tse-Tsung Liu

**Affiliations:** 1 Department of Nursing, College of Nursing, Tzu Chi University of Science and Technology, Hualien, Taiwan; 2 Department of Family Physician and Geriatrician, Mennonite Christian Hospital, Hualien, Taiwan

**BMC Geriatrics (2023) 23:749**

10.1186/s12877-023-04478-w

After publication of this article [1], the authors reported that the Chinese characters in Table 5 should be deleted. The original article [1] has been corrected.

## References

[1] Liao SJ, Fang YW, Liu TT (2023). Exploration of related factors of suicide ideation in hospitalized older adults. BMC Geriatr.

