# Supplementary material for: Exploration of related factors of suicide ideation in hospitalized older adults
Source: BMC Geriatr. 2023 Nov 16;23:749. doi: 10.1186/s12877-023-04478-w (PMC10655411; doi:10.1186/s12877-023-04478-w)
Supplement: Supplementary file 1 — Supplementary Material 1 [file 12877_2023_4478_MOESM1_ESM.docx]

**Demographic and medical characteristics**

| Gender | □Male □Female |
| --- | --- |
| Age | □65~74 □75~84 □85 year old above |
| Education level | □Recognize words □Elementary school |
|  | □Junior high school □Senior high school above |
| Living Status | □Alone □With spouse □With spouse and children |
| Economic Status | □Ample □Passable □Insufficient |
| Marital | □No spouse □Married |
| Perceived health | □Very bad □Not good □Good |

**BSRS-5 (Brief Symptom Rating Scale)**

**
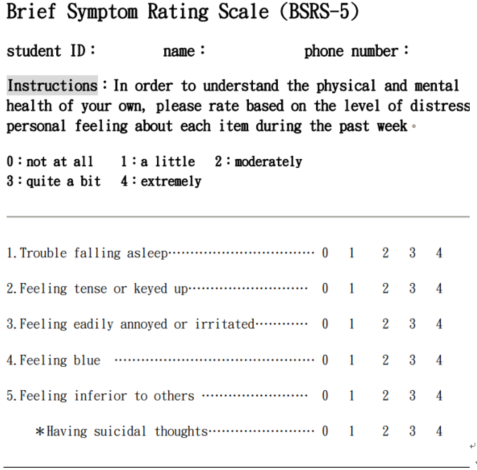
**

**Mini-Mental Status Examination (MMSE)**

**
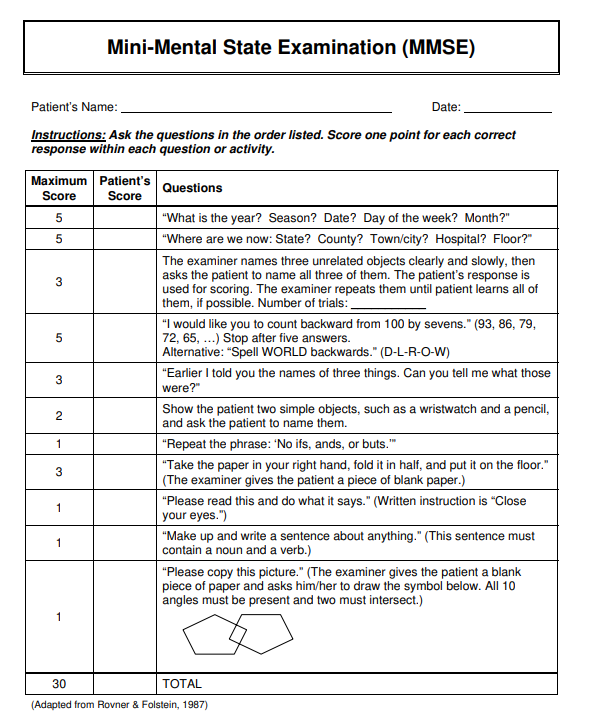
**

**World Health Organization Quality of Life-BREF (WHOQOL-BREF TW)**

| **Variable** | **1** | **2** | **3** | **4** | **5** |
| --- | --- | --- | --- | --- | --- |
| **Domain I Physical** |  |  |  |  |  |
| 1 Pain and discomfort |  |  |  |  |  |
| 2 Energy and fatigue |  |  |  |  |  |
| 3 Sexual activity |  |  |  |  |  |
| 4 Sleep and rest |  |  |  |  |  |
| 5 Sensory functions |  |  |  |  |  |
| **Domain II Psychological** |  |  |  |  |  |
| 6 Positive feelings |  |  |  |  |  |
| 7 Thinking, learning, memory and concentration |  |  |  |  |  |
| 8 Self-esteem |  |  |  |  |  |
| 9 Bodily image and appearance |  |  |  |  |  |
| 10 Negative feelings |  |  |  |  |  |
| **Domain III Level of independence** |  |  |  |  |  |
| 11 Mobility |  |  |  |  |  |
| 12 Activities of daily living |  |  |  |  |  |
| 13 Dependence on medicinal substances and medical aids |  |  |  |  |  |
| 14 Dependence on nonmedicinal substances (alcohol, tobacco, drugs) |  |  |  |  |  |
| 15 Communication capacity |  |  |  |  |  |
| 16 Work capacity |  |  |  |  |  |
| **Domain IV Social relationships** |  |  |  |  |  |
| 17 Personal relationships |  |  |  |  |  |
| 18 Practical social support |  |  |  |  |  |
| 19 Activities as provider/supporter |  |  |  |  |  |
| **Domain V Environment** |  |  |  |  |  |
| 20 Freedom, physical safety and security |  |  |  |  |  |
| 21 Home environment |  |  |  |  |  |
| 22 Work satisfaction |  |  |  |  |  |
| 23 Financial resources |  |  |  |  |  |
| 24 Health and social care: accessibility and quality |  |  |  |  |  |
| 25 Opportunities for acquiring new information and skills |  |  |  |  |  |
| 26 Participation in and opportunities for recreation/leisure activities |  |  |  |  |  |
| 27 Physical environment: (pollution/noise/traffic/climate) |  |  |  |  |  |
| 28 Transport |  |  |  |  |  |
| **Domain VI Spirituality/religion/personal beliefs** |  |  |  |  |  |
| Overall quality of life and general health perceptions |  |  |  |  |  |

Note: 1: Not at all; 2: A little; 3: A moderate amount; 4: Very much; 5: An extreme amount

**Geriatric Depression Scale Short Form, (GDS-SF)**

**
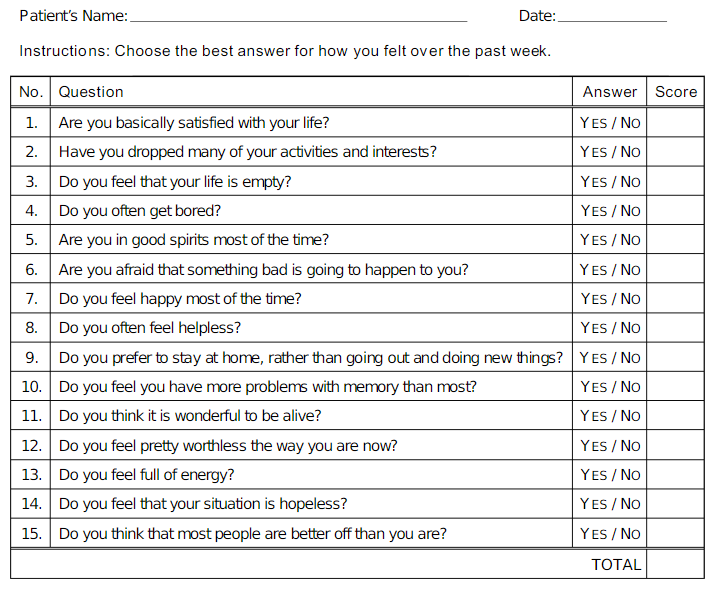
**

**Beck Scale for Suicide Ideation；BSS**

| **Variable**  **Variable** | **0** | **1** | **2** |
| --- | --- | --- | --- |
| 1.Wish to live * |  |  |  |
| 2.Wish to die* |  |  |  |
| 3.Reasons for living or dying |  |  |  |
| 4.Active suicide attempt |  |  |  |
| 5.Passive suicide attempt |  |  |  |
| 6.Duration of suicidal thoughts |  |  |  |
| 7.Frquency of ideation |  |  |  |
| 8.Attitude toward ideation |  |  |  |
| 9.Control over suicidal action |  |  |  |
| 10.Deterrents to attempt |  |  |  |
| 11.Reasons for attempt |  |  |  |
| 12.Specificity of planning |  |  |  |
| 13.Availability or opportunity |  |  |  |
| 14.Capability to carry out attempt |  |  |  |
| 15.Expectancy of actual attempt |  |  |  |
| 16.Extent of actual preparation |  |  |  |
| 17.Suicide note |  |  |  |
| 18.Final acts |  |  |  |
| 19.Deception and concealment |  |  |  |
| Total Score |  |  |  |
| 20.Frquency of suicide attempt |  |  |  |
| 21.Suicide attempt and wish to die |  |  |  |
