# Supplementary material for: Exploration of related factors of suicide ideation in hospitalized older adults
Source: BMC Geriatr. 2023 Nov 16;23:749. doi: 10.1186/s12877-023-04478-w (PMC10655411; doi:10.1186/s12877-023-04478-w)
Supplement: Supplementary file 2 — Supplementary Material 2 [file 12877_2023_4478_MOESM2_ESM.pdf]

The Mini-Mental State Exam

Patient______________________________ Examiner __________________________ Date___________

Maximum	Score	
		**Orientation**
5	( )	What is the (year) (season) (date) (day) (month)?
5	( )	Where are we (state) (country) (town) (hospital) (floor)?
		**Registration**
3	( )	Name 3 objects: 1 second to say each. Then ask the patient all 3 after you have said them. Give 1 point for each correct answer. Then repeat them until he/she learns all 3. Count trials and record. Trials __________
		**Attention and Calculation**
5	( )	Serial 7's. 1 point for each correct answer. Stop after 5 answers. Alternatively spell "world" backward.
		**Recall**
3	( )	Ask for the 3 objects repeated above. Give 1 point for each correct answer.
		**Language**
2	( )	Name a pencil and watch.
1	( )	Repeat the following "No ifs, ands, or buts"
3	( )	Follow a 3-stage command: "Take a paper in your hand, fold it in half, and put it on the floor."
1	( )	Read and obey the following: CLOSE YOUR EYES
1	( )	Write a sentence.
1	( )	Copy the design shown.

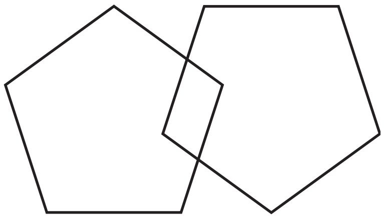

_____ Total Score

ASSESS level of consciousness along a continuum ___________

Alert Drowsy Stupor Coma

"MINI-MENTAL STATE." A PRACTICAL METHOD FOR GRADING THE COGNITIVE STATE OF PATIENTS FOR THE CLINICIAN. *Journal of Psychiatric Research*, 12(3): 189-198, 1975. Used by permission.
